# Disinfection and Isotonic Drinks’ Influence on Hardness and Color Stability of Ethylene-Vinyl-Acetate Copolymer Mouthguards Used in Martial Arts: An In Vitro Study

**DOI:** 10.3390/polym15081822

**Published:** 2023-04-08

**Authors:** Katarzyna Mańka-Malara, Marcin Szerszeń, Bartłomiej Górski, Gen Tanabe, Toshiaki Ueno, Elżbieta Mierzwińska-Nastalska

**Affiliations:** 1 Department of Prosthodontics, Medical University of Warsaw, 02-097 Warsaw, Poland; 2 Department of Periodontal and Oral Mucosa Diseases, Medical University of Warsaw, 02-097 Warsaw, Poland; 3 Department of Sports Dentistry, Tokyo Medical and Dental University, 113-8510, Tokyo, Japan; 4 Department of Sports Dentistry, Meikai University School of Dentistry, 350-0248, Saitama, Japan

**Keywords:** mouthguards, dental polymers, dental materials, disinfection, oral hygiene, combat sports, martial arts, occlusal splint

## Abstract

This in vitro study aimed to evaluate the hardness and color change of an ethylene-vinyl-acetate copolymer (EVA) material for mouthguards after exposition to different cleaning agent solutions and isotonic drinks. Four hundred samples were prepared and divided into four equinumerous groups (n = 100), in which there were 25 samples from each color of EVA (red, green, blue and white). The hardness, using the digital durometer, and the color coordinates (CIE L*a*b*), using the digital colorimeter, were measured before the first exposition and after 3 months of exposition to spray disinfection and incubation in the oral cavity temperature, or immersion in isotonic drinks. The values of Shore A hardness (HA) and color change (ΔE—calculated by Euclidean distance) were statistically analyzed using the Kolmogorov–Smirnov test, multiple comparison ANOVA/Kruskal–Wallis and appropriate post-hoc tests. Statistically significant changes in color and hardness between the tested groups were demonstrated after the use of agents predestined for disinfecting the surface of mouthguards on the tested samples. There were no statistically significant differences in color and hardness between the groups immersed in isotonic sport drinks potentially consumed by competitors practicing combat sports using mouthguards. Despite the changes in color and hardness after the use of disinfectants, the deviations were minor and limited to specific colors of the EVA plates. The intake of isotonic drinks practically did not change either the color or the hardness of the samples, regardless of the tested color of the EVA plates.

## 1. Introduction

A mouthguard is a protective polymeric device that covers the palate and occlusal surfaces of the teeth. During an injury, it protects the teeth, oral cavity structures and temporomandibular joint from trauma [1,2,3,4]. The American Standards for Testing Materials classifies mouthguards into three categories: stock, boil-and-bite and custom-made [5]. Custom mouthguards are prepared using thermoforming, traditional polymerization methods and thermal injection [6,7,8]. Ethylene vinyl acetate (EVA) is commonly used in mouthguard fabrication—both in boil-and-bite appliances, and those custom-made. It is a copolymer characterized by flexibility, elasticity and certified biocompatibility [9,10,11,12]. This thermoplastic polymer is made of separate monomers: ethylene and vinyl acetate (usually 1–40% by weight). EVA is a semi-crystalline polymer; its structure comprises an amorphous and crystalline component. The amorphous phase of EVA is represented by a macromolecule entanglement that lacks a three-dimensional organized and periodic structure. In contrast, the crystalline phase is distinguished by a three-dimensional organized and periodic structure of macromolecules folded one on the base of the other in the lamellar format. When the proportion of vinyl acetate (VA) in EVA falls, so does its damping capacity. In some circumstances, the stiffness and hardness of EVA increase according to the degree of crystallization. EVA is a macromolecule comprised of repeating units linked by covalent bonds. Its fundamental constituents are two monomers whose physical properties are determined by their size and molecular weight. Polymeric materials have a density range of 0.926 to 0.950 g/cm^3^. Among the most essential aspects of EVA is its elastic behavior, which is defined by a Young Modulus ranging from 15 to 80 MPa. [13,14,15]. Unfortunately, despite being the gold standard for protective splints, it has some disadvantages that should be considered. EVA copolymers are sensitive to repeated exposure to high temperatures and prolonged heating. There is a problem in thermoforming of achieving the optimal thickness of the appliance, necessary to provide adequate protection against the effects of trauma [10,16,17,18,19]. Loss of up to 25% of the thickness of the protector on the occlusal surface and 50% on the labial surface result in a reduction in its ability to dampen the force of the strike [20]. The use of color pigment in the EVA material influences its properties—the opaque materials have lower adhesive capabilities than clear or semi-transparent [21]. The literature also describes the hardening of ethylene vinyl acetate protectors and changes in the structure of the material under usage, temperature differences and pressure, which can affect the energy absorption properties [22]. Benli et al. [23] stated that EVA can be prone to wear and its long-term use is not recommended as it has an irregular surface which may increase plaque accumulation. As mouthguards should be used in various sport activities it should be also verified whether it is possible to use isotonic drinks having the protective splint in the oral cavity. Such beverages are used to refill the fluids lost during the exercise. Studies concerning the effect of isotonic drinks on dental materials and tooth structures have shown that they have erosive potential and decrease the hardness of restorative materials after immersion in tested solutions [24,25,26,27].

The optimal method of mouthguard sanitization is disinfection [28,29,30,31]. The disinfectant solution decreases the number of microorganisms isolated from its surface [29]. Unfortunately, there are still no conclusive guidelines comparing disinfectants. The changes in the porosity of the elastic polymeric material used in mouthguard fabrication may induce microbial colonization of its surface [28,31,32,33,34]. D’Ercole et al. [35] have shown that the use of intraoral protectors interferes with the oral environment—changing the pH of saliva, reducing its buffering capacity and increasing plaque accumulation and bleeding. The porous surface of the mouthguard contains opportunistic or pathogenic bacteria and fungi, while its roughness, which appears after too long and unhygienic use, can cause minor soft tissue injuries [23]. The damage of such an appliance can cause the migration of microorganisms into the pterygoid plexus [23,28]. A microbiological analysis of intraoral protectors used by 62 American football players revealed the presence of 356 bacillus strains, 22 yeast-like fungi and 107 molds. Ifkovits et al. [36] found that 89.9% of mouthguards used by athletes are defective due to their incomplete coverage of the labial surface of the teeth, improper interocclusal contacts and destruction of the splint surface. Glass et al. [30] recommended replacing the mouthguard with a new appliance once surface porosity appears. They also indicated that it should be perceived as a therapeutic device—it should be replaced after the damage of its surface, 14 days of regular use or in cases of oral mucosal lesions or respiratory infection [31]. Because of the risk of cross-infection, they also considered whether protective occlusal splints should be disposed of after single use [28]. Due to economic reasons and lack of awareness, this is still unlikely—research has shown that 20% of athletes never replace their mouthguard and 85.5% of athletes choose tap water rinsing as the primary method of protective splint cleaning [37]. Disinfection, recommended in the literature, is currently used by only 2.7% of mouthguard users. It is necessary to choose a disinfectant that does not damage the surface of a protective splint and to introduce guidelines for mouthguard maintenance [38,39]. The aim of this research was to compare the influence of different disinfectants and isotonic drinks on the hardness and color stability of EVA used in mouthguard fabrication. The results of the study could contribute to providing guidelines for correct mouthguard maintenance for their users.

## 2. Materials and Methods

### 2.1. Preparation of Samples

The test material used in this study was ethylene vinyl acetate plates (EVA, Drufosoft, Dreve, Unna, Germany) with standard dimensions of 1200 mm in diameter and 3 mm in thickness. There were 400 EVA samples prepared, 100 for each evaluated color (red, green, blue, and white), cut from rounded plates using a scalpel in the form of cuboids 10 mm × 10 mm × 3 mm. Four tested groups were created—in each, there were 100 samples (25 from each color).

### 2.2. Sample Disinfection Protocol

**Figure 1:**
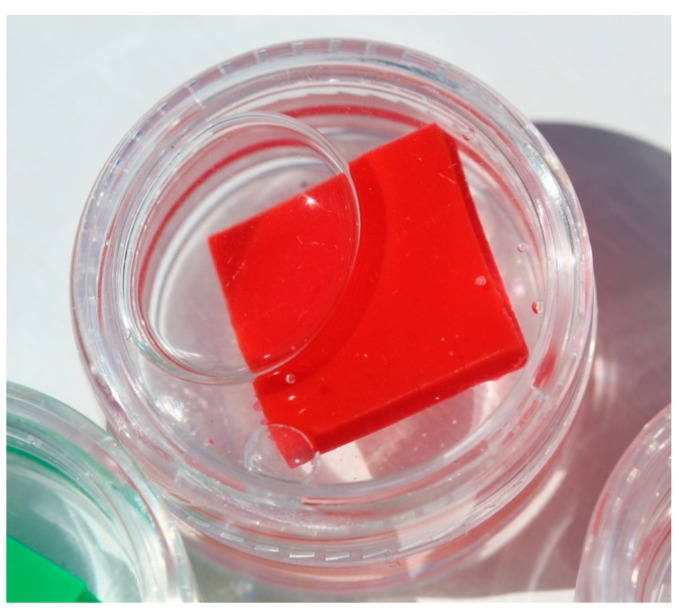
EVA sample in a separate container prepared for incubation.

In this part of the study, 320 samples were tested, 80 from each color of EVA. Three times a week, all samples were put in small (3 mL), separate containers (Figure 1), into the Natrium Chloratum (NaCl) 0.9% solution (Braun, Meisunger, Germany; according to the manufacturer’s data with a pH of 4.5–7) changed for each exposition, for an hour into the laboratory incubator (Elkon CL-65, Rybnik, Poland) set on temperature 36.6 °C. The frequency and duration of incubation were based on previous research—most martial arts practitioners train about three times a week and the mouthguard is placed in the oral cavity for about an hour as an athlete does not use it during the initial part of the training [40]. After each incubation, the tested samples were disinfected and put into ventilated containers until the next exposition. The first group was disinfected using the spray containing chlorhexidine (CHX) digluconate 0.12%, and cetylpirydynium chlorite 0.05% (Perio-AID, Dentaid, Barcelona, Spain); the second—Safe JAWZ spray (Safe Jawz, Aldrige, UK) dedicated to mouthguard disinfection; the third—spray with ethyl alcohol (Bioseptol AMF, Chełmża, Poland). The fourth group was the control—the samples were incubated in the NaCL but they were not disinfected. The pH of the disinfectants measured using the calibrated ph-Meter PH-100ATC (Volcraft, Hirschau, Germany) was pH 5.23 for Perio-AID spray, pH 4.74 for Safe JAWZ spray, and pH 4.47 for Bioseptol AMF spray. The pH of saline used in the study was 5.5. The hardness and color were measured before the first exposition and after 3 months. The total effective exposition period was 36 h.

### 2.3. Protocol for Immersion of Samples in Isotonic Drinks

**Figure 2:**
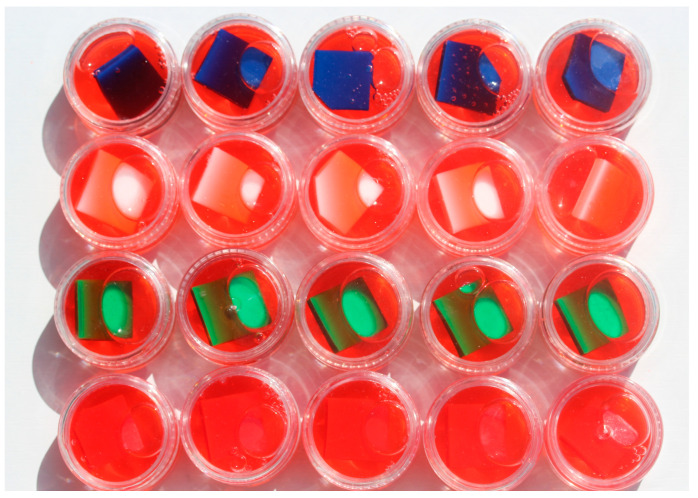
EVA samples in separate containers in isotonic drink (Oshee Red Orange Flavour).

There were 80 EVA samples tested—20 for each evaluated color. The number of samples was lower in each group than in the disinfection protocol because in this case the exposure was continuous, and samples were immersed in tested solutions, so the time and direct effect was assumed to be of greater intensity—the number of samples was coordinated with the assumed statistical tests. Four tested groups were created—in each, there were 20 samples (5 from each color). Samples were placed in small, closed containers. In three groups, they were immersed in isotonic drinks—Oshee Red Orange Flavour, Oshee Lemon Flavour, and Oshee Multifruit Flavour (Oshee Poland Sp. z o.o., Kraków, Poland) istotonic drinks were used in the evaluation. The list of ingredients of the isotonic drinks is given in Table 1. Samples from the fourth control group were immersed in spring water (Żywiec Zdrój, Danone, Paris, France). The pH of the isotonic drinks measured using calibrated ph-Meter PH-100ATC (Volcraft) was 2.99 for Oshee Red Orange Flavour, 3.22 for Oshee Lemon Flavour and 2.94 for Oshee Multifruit Flavour. All samples remained in tested solutions continuously for 3 months (Figure 2). The hardness and color were measured before the exposition and after 3 months of immersion. The long-term immersion protocol has been used in colorimetric studies of denture-based materials [41,42]

### 2.4. Hardness and Color Change Measurements

The digital durometer (LX-A, Huatec Group Corporation, Beijing, China), with constant load test stand (TI-D, Sauter -KERN & SOHN GmbH, Ballingen, Germany), using the Shore A scale was used to measure the hardness of all samples. For the color comparison, the digital colorimeter (ColorReader, Datacolor AG Europe, Switzerland) compliant with the standards adopted by the International Commission of Illumination for colorimetric tests, including a 10° observational angle, D65 luminant with built-in light source in the form of six light diodes. The data acquired with the device were sent by a wireless Bluetooth interface, and were analyzed using the three-dimensional measurement system—CIE L*a*b* color space where values represent: L*—the object clarity (from black, 0, to white, 100); a*—the quality of red (a > 0) or green (a < 0); and b*—the quality of yellow (b > 0) or blue (b < 0). The obtained values were compiled using descriptive statistics (mean and standard deviation—SD). The differences between the groups were calculated using the ∆E parameter according to the distance formula. ∆E is the Euclidean distance between two colors in the space with the assumption that they are both described in the same space and expressed as a number. All measurements were made by the same operator, in the same research and control station, in the same lighting conditions (dark room).

### 2.5. Statistical Analysis

The statistical analysis of obtained data was performed with PQStat Software (v.1.8.4.142, PQStat Software, Poznań, Poland). Descriptive statistics including means and standard deviations were performed. Due to the knowledge of the mean values and the standard deviation, the normal distribution of data was verified using Kolmogorov–Smirnov tests. ANOVA or Kruskal–Wallis tests and then post-hoc tests were performed in groups with statistically significant differences. In order to compare the individual groups with each other, the Sheffe testing was used in the case of the ANOVA test due to its conservative nature. In the case of contraindications to parametric testing, failure to meet the requirement of equality of variance (in the Brown–Forsythe test), post-hoc Dunn–Bonfferoni was used. The level of significance for tests was set at *p* < 0.05.

## 3. Results

### 3.1. Influence of Cleaning Agents on Color and Hardness Changes of EVA

Regardless of the disinfection spray used, the color of the samples changed during the test. The average color change for all EVA colors in the test procedure treated with disinfectants was almost twice higher than in the control group (test groups 1.64 (±0.81), control group 0.92 (±0.45)). Considering the division due to the color of the EVA plates, the highest value was calculated for the green samples and was 8.08 for the group immersed in the CHX solution. The greatest color changes were caused by the CHX-containing solution, apart from samples made of white material. Changes in the values of the ∆E calculation components in the form of the CIE L* component were also most noticeable, which may indicate a tendency to bleach samples under the influence of Perio-AID.

**Figure 3:**
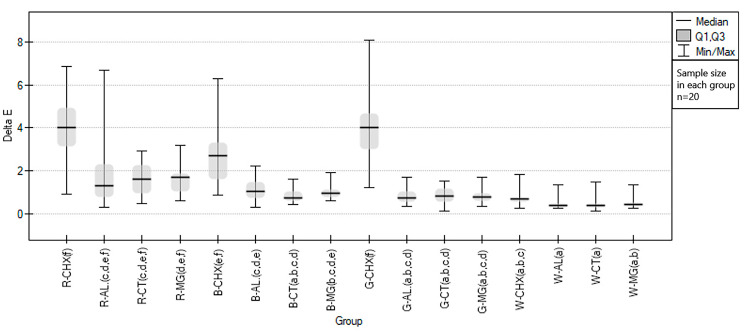
Graphical representation of the results of statistical analysis of the color changes of EVA samples under the influence of disinfecting materials, divided into groups (*n* = 20). The lowercase letters in brackets after the group code indicate homogeneity between the groups in post-hoc Dunn–Bonferroni testing.

Testing with Kruskal–Wallis was performed, reaching statistical significance (*p* < 0.000001), which was the basis for further proceedings in the form of post-hoc tests (Dunn–Bonferroni)—the statistical results are presented in a graphical form in Figure 3, see Table 2.

**Figure 4:**
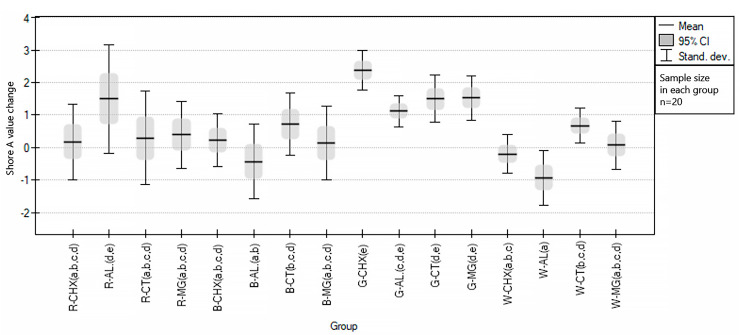
Graphical representation of the results of statistical analysis of the hardness of EVA samples under the influence of disinfecting materials, divided into groups. The same lowercase letters in brackets after the group code indicate homogeneity between the groups in post-hoc Sheffe testing.

The part of the study concerning changes in the hardness of samples under the influence of disinfectants showed that, despite the changes noted, the fluctuations of the HA index were at a very low level. The mean change in hardness regardless of the disinfectant in the test and control groups was 0.5 (0.91) and 0.8 (0.92), respectively. The variation visible in some groups may be the effect of the heterogenous surface of the samples after exposition or covering the surface of the samples with sprayed disinfectant. When comparing the groups in terms of the impact of specific disinfectants on their hardness, the ANOVA statistical test was used, obtaining a statistically significant *p* < 0.000001. Post-hoc tests (Sheffe testing) showed which groups differed from each other and which showed homogeneity; this is illustrated in Figure 4.

The maximum unit specimen hardness change was recorded for the group of red samples disinfected with Safe JAWZ spray (R-AL group) and was 4 HA, while the largest average change in hardness was recorded for the group of green samples disinfected with Perio-AID and was 2.38 (0.60).

### 3.2. Influence of Sport Drinks on Color and Hardness Changes of EVA

**Figure 5:**
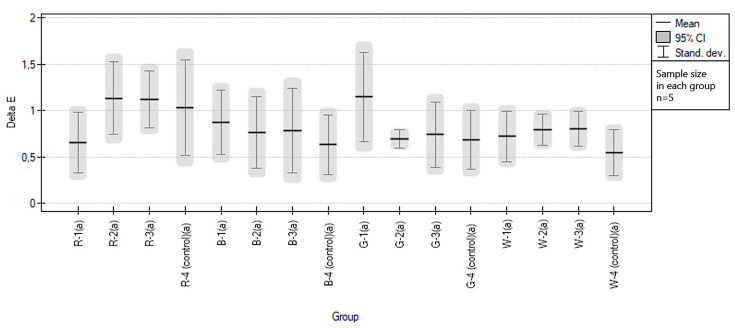
Graphical representation of the results of statistical analysis of the color changes of EVA samples under the influence of sports drinks, divided into groups. The same lowercase letters in brackets after the group code indicate homogeneity between all groups.

In this part of the study, we compared the impact of three types of sports drinks in the form of isotonic fluids of one brand. The color change oscillated between 0.24 and 1.91. The lowest ∆E value was recorded for the red EVA sample and the highest for the green EVA sample. In comparison, in the control groups, the lowest and highest recalculated ∆E values were 0.23 (for the white EVA sample) and 1.78 (for the red EVA sample), respectively. Regardless of the color of the samples and drink, average changes of color were 0.85 (±0.35) for study groups and 0.72 (±0.38) for control. In statistical calculations, after meeting the requirements of uniformity of distribution (Kolmogorov–Smirnov tests) and variance (Brown–Forsythe test *p* = 0.850321), an ANOVA analysis was performed, obtaining the value *p* = 0.117293 (*p* > 0.05), indicating no statistically significant differences between the groups. Homogeneity was maintained between all groups in the study. Mean values with standard deviations for individual groups are presented in Figure 5, see Table 3.

The average hardness changes of EVA samples under the influence of isotonic drinks were higher than in the case of disinfectants. Across the study, the groups changed an average of 2.78 (±1.31) for the tested groups and 2.40 (±0.38) for the control groups. Some samples (blue and white EVA) did not change their hardness at all, while the highest change in hardness recorded in the study was for the white sample—6.5 HA. To compare, the highest value recorded in the control groups alone was 5 HA. A null hypothesis was adopted that the variances in individual groups were not statistically significantly different (Brown–Forsythe *p* = 0.854758). Then, an ANOVA test was performed, which showed that there were no statistically significant differences between the groups, reaching the value of *p* = 0.365338.

**Figure 6:**
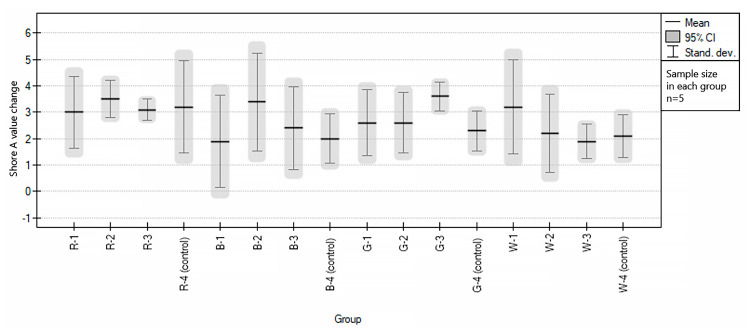
Graphical representation of the results of statistical testing of the hardness of EVA samples under the influence of sports drinks, divided into groups.

Figure 6 graphically presents the mean values with deviations for individual groups. The study showed the homogeneity of hardness changes between all groups, regardless of the immersion agent used in this part of the study. Throughout the study, the average change in the hardness of the samples regardless of the EVA color tested was 2.69 (1.28) HA.

## 4. Discussion

The use of disinfection sprays causes hardening of the EVA samples in a very small range. Changes are so subtle that they remain within the accuracy level of the used device, and it could not be stated that it would be clinically noticeable to such an extent. Although the degree of changes occurring in 3 months does not seem to be clinically important, cumulated changes after longer usage could lower the comfort of the user and reduce energy absorption capacities—according to the literature, most athletes do not change the used appliance at regular intervals [37]. The hardening of the material may influence the performance of the mouthguard [31,37]. The main feature of the appliance is the protection of teeth and oral cavity structure from injury. Previously conducted studies confirmed that the energy-absorbing qualities of the material used in their fabrication are correlated with the hardness of the material [3,37]. Additionally, softer mouthguards are perceived as more comfortable by users [43]. Current results show also that the use of disinfectant containing 0.12% CHX and 0.05% cetylpirydynium chloride may influence the EVA color. Evaluation of the type of color changes revealed that there was an increase in the lightness value (L*) defined from black at 0 to white at 100, which explains why changes were not significant for white samples. The highest color changes were noted in red samples, which means that there may be differences in color stability between different material colors. Statistically significant changes were noted in green material samples. However, we cannot exclude that if the time of exposure was further extended, significant changes could be observed also in red and blue samples, and further research should include evaluation after longer exposure to tested disinfectants. The presented study showed also that long-term immersion in isotonic drinks did not cause statistically important changes in the color of the samples. However, there was a slight increase in the mouthguard hardness after immersion in all solutions, without statistically important differences between test groups, which potentially may result in deterioration of the protective function. The current study involves evaluating only one type of commercially available EVA plate. Unfortunately, the manufacturer does not provide the specific composition of the material. The VA content in commonly available EVA ranges between 1 and 40 wt.-%. [12]. Its constitution strongly influences the properties—at 40 wt.-% VA it is soft polymer with a broad melting point of approximately 25–55° [44,45,46]. The use of EVA with different VA content could influence the achieved results. Additionally, EVA retains high flexibility and material diffusivity even at very low temperatures. The disinfection of samples after incubation in 36° may influence the effect of disinfecting sprays on the material composition. However, such conditions resemble real-life application, when athletes sanitize the splint after removing it from the oral cavity. Due to the specific application of mouthguards, they are usually not exposed to the hot temperatures associated with food or beverage intake.

The hardening of EVA mouthguards have been previously evaluated by Kuwahara et al. [22]. They conducted ^13^C NMR measurements, Pulse NMR measurements, DSC measurements, repeated compression and thermal cycle experiments, as well as compressive stress–strain measurements, on samples made of the same EVA material (Drufosoft, Dreve, Germany). Temperature fluctuations and repeated pressure changes affect the protective ability of protective splints. EVA is a macromolecule with entangled polymer chains comprising crystalline and amorphous phases [46,47]. The changes in the crystallinity of EVA during routine use explain the hardening that occurs after a period of usage. The increase in the relative amount of the crystalline phase may be primarily attributed to temperature fluctuations and repeated changes in pressure [22]. The change in the vinyl acetate content in the copolymer changes the material properties—the VA groups inhibit crystallization of the polyethylene chain segments, enabling variation in the softness and crystallinity without the addition of low molecular weight plasticizers [12]. In the EVA, apart from the immobile orthorhombic phase and monoclinic crystalline phases, a third crysthalline phase SOCP was detected via solid state NMR and DSC. Such a phase forms during room-temperature aging and melts at temperature higher than that on heating [47]. Conducted research also showed that simulated usage with disinfection had a statistically significant influence on the hardness of EVA mouthguards. Clinically, EVA mouthguards have increased roughness and significant changes of the surface after intraoral application [48].

The chemical substances used in the current study have previously been used in mouthguard disinfection. Riberio et al. [48] conducted a randomized clinical trial on the application of 0.12% chlorhexidine spray and its influence on contamination and surface roughness of vacuum-pressed EVA mouthguards. The use of disinfecting spray significantly reduced bacterial cell viability. All mouthguards had significantly increased surface roughness after being evaluated for 15 days of usage, regardless of the sanitization method applied—no differences were found between the control and experimental groups. The use of chlorhexidine on mouthguards was also studied by D’Erkole et al. [49] who showed that it inhibits the growth of microbial species and improves pH value. Unfortunately, the influence of such disinfectant on the copolymer was not evaluated. Wood et al. [50] proposed coating EVA with chlorhexidine, gradually released from the material. However, the long-term use of this additive is somewhat questionable due to its cytotoxicity to human gingival fibroblasts, possible tooth discoloration, taste changes or even the risk of anaphylactic reaction [50,51,52,53]. In the current research, the evaluated spray also had cetylpyridinium chloride—used as a disinfectant in dentistry in concentrations of 0.05% to 0.75%—and its addition may influence the results [54,55]. Whitaker et al. [56] described the possibility of using hand sanitizer for on-site mouthguard disinfection. The main active ingredient of the applied disinfectant was alcohol, which evaporates rapidly and is not toxic with respect to soft tissues. According to the literature, chemical disinfecting agents containing alcohol affect the flexural strengths of the non-crosslinked denture base resins and affect the interphase region between the polymethyl metacrylate polymer bead and the polymer matrix [57,58]. Further research should investigate the influence of its application on the EVA copolymer. One of the sprays used in the current study was dedicated to mouthguards disinfection. Previous studies on the antimicrobial effects of MG disinfectant sprays have proved their effectiveness [59,60]. Unfortunately, the manufacturer does not provide its composition; therefore, the influence of its active ingredients cannot be discussed. The lack of this information should be considered as a limitation of this study.

The sanitization of restorations made of EVA was also studied by Ogawa et al. [59]. The study consisted of inserting 5 mm × 20 mm × 1 mm plates of Erkoflex (Erkodent, Pfalzgrafenweiler, Germany) material into the oral cavity of seven healthy volunteers who had previously undergone professional mechanical tooth cleaning. The samples were rinsed after removal with sterile water for 10 s, then cleaned with a toothbrush for 5 min or with a disinfected mouthguard (Mouthguard Cleaner, Earth Chemical Co., Tokyo, Japan). After sanitization, they were stored at room temperature in ventilated containers or sealed tubes for 0, 1, 2, 3, 7, 14, 21 or 28 days. The use of ventilated containers resulted in a reduction in live isolated bacteria—after two days, all samples stored in such a manner were free of their presence. Closed containers allowed bacteria to survive for up to 14 days. The authors emphasized that although rinsing with sterile water and brushing does not kill bacteria, it is effective due to the attenuation of bacterial colonization. Nagai et al. [61] proposed introducing a bioactive filler of pre-reacted glass-ionomer (S-PRG) into the EVA. Analysis of the properties of such material showed the activity against the *Streptococcus mutans* and *Porphyromonas gingivalis*, while showing no cytotoxic effects. However, the introduction of the particles slightly changed the physical properties of the samples. Evaluating the effect of decontamination on the surface of the materials used in mouthguard fabrication showed that only samples made of hybrid acrylic (Impak, Vernon-Benshoff Comp., Pitsburgh, PA, USA), disinfected with a spray dedicated to mouthguards, maintained a homogeneous surface [62]. A protector made via the traditional polymerization technique from this material can be subjected to regular disinfection by the user. The surface of EVA was uneven and contained grooves and cracks in all types of sanitization methods. Similar doubts concerning the uneven structure of this material were stated by Benli et al. [23]. In the current study, samples of EVA were not thermoformed before the research, while previous studies showed that it could create damage to the surface [21], and the achieved results could be different. There are only three disinfectants tested—to compare new solutions, it would be beneficial to conduct equivalent research using new products. Additionally, in the study all EVA plates were from only one manufacturer to maintain consistency and compare the colors—however, the use of other materials could also provide different results. The use of different colors of EVA materials was also used in the research conducted by Del Rossi et al. [21]. Although they did not compare their color stability, they discovered that they behave differently during thermoforming—the dark-color plates enabled one to achieve superior adaptation and produce more firmly fitting mouthguards. Despite the aesthetic aspect of the protective splint color, it is also an important factor in the visibility of a splint—the study by Wilkinson and Powers [63] showed that yellow mouthguards were visible at almost twice the distance of the clear protector. Almeida et al. [64] showed that the color and thickness did not influence the surface roughness.

Storage conditions and disinfection can influence the properties of prosthodontics materials [65,66]. However, there are some limitations of the applied methodology which should be considered—the simulation of the oral cavity environment by increased temperature and immersion in NaCl is simplified and may not perfectly reflect in vivo conditions. The temperature in the oral cavity and saliva composition during exercise changes. With the increase in the temperature in the oral cavity, the polymer material comes closer to the glass transition temperature (TG), leading to deformation at lower stress levels [67,68]. D’Ercole et al. [69] demonstrated that footballers had a statistically higher salivary microbial load than in boys who did not practice any sport. Additionally, during training time, a statistically significant decrease in S-IgA concentration occurred. As the level of CO_2_ increases in the blood during a sports performance, the salivary pH decreases [70]. Sport activity is characterized by greater salivary function and intense physiological response. Due to the difficulties of fabrication predictability and cleaning maintenance, it should be considered whether mouthguards could be made using different polymers. Protectors made with hybrid acrylic are positively evaluated by users and physicians [9,43,71]. In addition, these materials have adequate impact energy absorption properties [21]. Currently, new methods for mouthguard fabrication are being introduced—the use of digital techniques could perhaps allow one to 3D print a new appliance without the need for another impression, opening the possibility for more frequent replacement. Of course, the new methods and materials used will require thorough testing, but preliminary reports are promising [72,73,74]. Li Z et al. [72] published preliminary results evaluating the use of CAD/CAM methods in the design of protective splints from PEEK, indicating that such mouthguards had favorable clinical properties. Liang et al. [73] evaluated the possibility of using 3D printing for protective splints. However, despite the advantageous properties of such materials, the most used polymer for mouthguard fabrication is still EVA and thus the current recommendations should be created for this material.

Prolonged exercise causes the loss of body fluids associated with elevated sweat rates [75,76]. Dehydration greater than 3–5% of total body water may reduce cardiac output, increase perceived exertion and impair thermoregulatory function, muscle blood flow and endurance exercise performance [77,78,79,80]. The use of isotonic sports drinks is also recommended in martial arts, as it promotes rehydration during the training session and reduces post-training proteinuria [81]. According to Erdemir et al. [24], the immersion of restorative materials in sports and energy drinks causes a change in their surface hardness. After 6 months of exposition, the softening of all restorative materials was observed, which was probably caused by water absorption and reducing the frictional forces between the polymer chains [25,82,83]. The environment of the oral cavity is difficult to replicate by in vitro experimental conditions—saliva has protective effects in moderating the extent of wear, abrasion and fatigue of restorative materials by interfering with the hydrolytic degradation process. The use of artificial saliva may be considered in material studies to mimic oral conditions [84,85,86]. However, the natural changes in saliva composition are still hard to duplicate in the research study, and solutions used in the literature have a different composition which may influence the achieved results [87]. In the conducted study, the NaCl used after each exposition was used as a control in disinfection, and water in the isotonic drinks part. This simplification, although improving the repeatability of the study, may be considered also as its limitation, as the use of different solutions could provide different results. Additionally, the long-term immersion does not replicate the real intake of isotonic drinks during martial arts training. However, the true exposition is difficult to estimate, as many mouthguard users do not maintain the correct hygiene of the used appliance and do not replace it regularly [37]. Thus, the remains of isotonic drinks could stay longer on the surface of the splint than actual intake during training. To confirm that the isotonic drinks do not worsen the properties of mouthguard material and evaluate the long-term influence and verify the tendencies, the conducted study involved the long-term immersion protocol. As a result, in real-life exposure the described effects would probably be observed in a longer period of time. Research concentrating on the durability of mouthguard materials is necessary, as there are reports on the possibility of using a biosensor in mouthguards, monitoring the parameters of saliva [88]. Arakawa et al. [88,89] described the possibility of embedding a sensor for monitoring saliva glucose. The sensor evaluating the saliva composition should be embedded in a material that is durable, and resistant to external factors.

## 5. Conclusions

Under the conditions of the current research, it can be concluded that exposure to disinfectant sprays causes 0.71% increase of hardness of EVA material. The application of spray containing 0.12% chlorhexidine digluconate and 0.05% cetylpirydynium chloride changes the color of EVA samples, and therefore its influence on the physical properties should be further evaluated—the change in the EVA color may be caused by changes in the copolymer structure. Exposure to the isotonic drinks does not cause change in EVA color, but immersion in all evaluated fluids caused an increase in mouthguard hardness. Further research should be conducted to verify whether such changes are clinically important and repeatable in in vivo conditions. The recommended disinfectant spray for mouthguard sanitization should be based on alcohol. The commercially available disinfectant spray does not negatively influence the color of the protective appliance. Athletes should be informed that isotonic drink intake during training with a mouthguard and the obligatory disinfection of the appliance may increase its hardness, and therefore its condition should be monitored by a dentist.

## Figures and Tables

**Table 1:** The ingredients of the isotonic drinks used in this study.

Oshee Red Orange Flavour	Oshee Lemon Flavour	Oshee Multifruit Flavour
water	water	water
glucose	glucose	glucose
citric acid	maltodextrin	maltodextrin
sodium citrate	citric acid	citric acid
potassium citrates	trisodium citrates	sodium citrates
flavours	tripotassium citrates	potassium citrates
potassium sorbate	flavours	flavours
potassium benzoate	potassium sorbate	potassium sorbate
gum arabic	potassium benzoate	potassium benzoate
glycerol esters of wood rosins	gum arabic	gum arabic
sucralose	glycerol esters of wood rosins	glycerol esters of wood rosins
Allura Red AC	aspartame	aspartame
niacine	acesulfame K	acesulfame K
vitamin B	guinoline yellow	brilliant blue FCF
biotin	niacine	niacine
	vitamin B	vitamin B
	biotin	biotin

**Table 2:** Abbreviations for groups in disinfection protocol.

Abbreviation	Group Description
R-CHX	red samples disinfected with Perio-AID
R-AL	red samples disinfected with Bioseptol AMF
R-CT	red samples control group (without disinfection)
R-MG	red samples disinfected with Safe JAWZ spray
B-CHX	blue samples disinfected with Perio-AID
B-AL	blue samples disinfected with Bioseptol AMF
B-CT	blue samples control group (without disinfection)
B-MG	blue samples disinfected with Safe JAWZ spray
G-CHX	green samples disinfected with Perio-AID
G-AL	green samples disinfected with Bioseptol AMF
G-CT	green samples control group (without disinfection)
G-MG	green samples disinfected with Safe JAWZ spray
W-CHX	white samples disinfected with Perio-AID
W-AL	white samples disinfected with Bioseptol AMF
W-CT	white samples control group (without disinfection)
W-MG	white samples disinfected with Safe JAWZ spray

**Table 3:** Abbreviations for groups in isotonic drink protocol.

Abbreviation	Group Description
R-1	red samples immersed in Oshee Red Orange Flavour
R-2	red samples immersed in Oshee Lemon Flavour
R-3	red samples immersed in Oshee Multifruit Flavour
R-4 (control)	red samples immersed in water (control group)
B-1	blue samples immersed in Oshee Red Orange Flavour
B-2	blue samples immersed in Oshee Lemon Flavour
B-3	blue samples immersed in Oshee Multifruit Flavour
B-4 (control)	blue samples immersed in water (control group)
G-1	green samples immersed in Oshee Lemon Flavour
G-2	green samples disinfected with Bioseptol AMF
G-3	green samples immersed in Oshee Multifruit Flavour
G-4 (control)	green samples immersed in water (control group)
W-1	white samples immersed in Oshee Red Orange Flavour
W-2	white samples immersed in Oshee Lemon Flavour
W-3	white samples immersed in Oshee Multifruit Flavour
W-4 (control	white samples immersed in water (control group)

## Data Availability

Data presented in the study are available on reasonable request from corresponding authors of this article.
